# COVID-19 and Obesity: An Epidemiologic Analysis of the Brazilian Data

**DOI:** 10.1155/2021/6667135

**Published:** 2021-05-05

**Authors:** Diego Assis Gonçalves, Victória Ribeiro, Ana Gualberto, Fernanda Peres, Michaela Luconi, Jacy Gameiro

**Affiliations:** ^1^Department of Parasitology, Microbiology and Immunology, Federal University of Juiz de Fora, Juiz de Fora, Brazil; ^2^Department of Experimental and Clinical Biomedical Sciences “Mario Serio”, University of Florence, Florence, Italy; ^3^Department of Pharmacology, Federal University of São Paulo, São Paulo, Brazil

## Abstract

Brazil has the second highest number of deaths due to COVID-19. Obesity has been associated with an important role in disease development and a worse prognosis. We aimed to explore epidemiological data from Brazil, discussing the potential relationships between obesity and COVID-19 severity in this country. We used a public database made available by the Ministry of Health of Brazil (182700 patients diagnosed with COVID-19). Descriptive statistics were used to characterize our database. Continuous data were expressed as median and analyzed by the nonparametric tests Mann–Whitney or one-sample Wilcoxon. The frequencies of categorical variables have been analyzed by chi-square tests of independence or goodness-of-fit. Among the number of deaths, 74% of patients were 60 years of age or older. Patients with obesity who died of COVID-19 were younger (59 years (IQR = 23)) than those without obesity (71 years (IQR = 20), *P* < 0.001, and *η*^2^ = 0.0424). Women with obesity who died of COVID-19 were older than men (55 years (IQR = 25) vs. 50 (IQR = 22), *P* < 0.001, and *η*^2^ = 0.0263). Furthermore, obesity increases the chances of needing intensive care unit (OR: 1.783, CI: 95%, and *P* < 0.001), needing ventilatory support (OR: 1.537, CI: 95%, and *P* < 0.001 and OR: 2.302, CI: 95%, and *P* < 0.001, for noninvasive and invasive, respectively), and death (OR: 1.411, CI: 95%, and *P* < 0.001) of patients hospitalized with COVID-19. Our analysis supports obesity as a significant risk factor for the development of more severe forms of COVID-19. The present study can direct a more effective prevention campaign and appropriate management of subjects with obesity.

## 1. Introduction

The COVID-19 (coronavirus disease 2019) has been declared as a pandemic in March 2020 by the World Health Organization [1]. The disease is caused by severe acute respiratory syndrome coronavirus 2 (SARS-CoV-2), which belongs to the Coronaviridae family. To date, more than 70 million people worldwide have been confirmed to be infected, with 1,599,704 deaths [2–4]. Currently, Brazil is the country with the second highest number of fatalities and third highest total cases, 180,437 and 6,836,227, respectively [2, 5]. More than 50,000 new cases keep on being reported per day in the country [5], and a high number of adults are considered at risk for severe COVID-19 in Brazil [6].

Since the beginning of the pandemic, studies have shown that the number of patients requiring intensive care, as well as the number of deaths, is greater among individuals over 60 years of age. Furthermore, these studies showed that some preexisting diseases can be considered risk factors, such as hypertension, diabetes, and cardiovascular disease [7–9]. Although obesity has not previously been reported among the main comorbidities related to COVID-19, it has an important role in the disease development and a worse prognosis [10–12]. This discussion proves to be very relevant, especially in the current pandemic scenario, since COVID-19 has been strongly affecting Brazil and the USA, both being populous countries where the obesity rate in adults reaches 22.1% and 36.2%, respectively [13].

Obesity has recently been recognized by the World Obesity Federation as a chronic disease and defined in Western countries as an excessive fat accumulation in people with body mass index (BMI) equal to or higher than 30 kg/m^2^ [14, 15]. Currently, it represents a health, social, and economic emergency with an exponential epidemic growth: in 2016, approximately 2 billion adults worldwide were overweight and 650 million were with obesity [14, 16].

The etiology of obesity is multifactorial, and the imbalance among consumption and expenditure calories is essential for obesity development [17]. Biologically, obesity is a chronic low-grade inflammatory state that alters metabolic and immunological pathways resulting in altered functions of the adipose tissue and also affecting other organs [18]. In this context, large amounts of cytokines and chemokines are released, and cells that produce proinflammatory molecules are recruited, culminating in unbalanced immune responses [18, 19].

The present study aims to explore epidemiological data from Brazil, discussing the potential relationships between obesity and COVID-19 severity in this country.

## 2. Methods

### 2.1. Data Sources

For the analysis performed in this study, we used a public database (SARS 2020—Severe Acute Respiratory Syndrome Database—including data from COVID-19), registered in the Influenza Epidemiological Surveillance Information System, SIVEP-Gripe (Sistema de Informação de Vigilância Epidemiológica da Gripe), and made available by the Ministry of Health of Brazil [20]. Since 2009, due to the Influenza A (H1N1) pandemic, the Ministry has been developing a SARS monitoring system. In 2020, the data from the pandemic COVID-19 were incorporated into the system. These data are regularly updated on the website, and we used those available by July 7, 2020. From the data provided, we used information about age, sex, presence/absence of comorbidities (here, we considered the most frequent comorbidities: cardiovascular disease, diabetes, chronic kidney disease, chronic neurological disease, pneumopathy, obesity, immunosuppression, and asthma), case classification (SARS by influenza, SARS-CoV-2, or other types of the virus), patient's death or cure, and if there was a need for admission to the intensive care unit and (non)invasive ventilatory support. Only confirmed cases of COVID-19 were considered for this study as inclusion criteria.

We created two additional variables, hypertension and cancer, from a column identified as “other risk factors” containing several other diseases listed. For this reason, for these two diseases, only the “yes” (presence) information was indicated, and due to this limitation, they were excluded from some statistical analyses.

This work was developed using a public database that does not identify research participants; therefore, approval by an ethics committee is unnecessary.

### 2.2. Statistical Analysis

We used descriptive statistics to characterize our database. Due to the presence of outliers and the nonnormality of the distribution (assessed by the Kolmogorov–Smirnov test), the continuous variable (age) was expressed as median (interquartile range (IQR)) and analyzed by the nonparametric tests Mann–Whitney test or one-sample Wilcoxon test. The frequencies of categorical variables were analyzed by chi-square tests of independence or of goodness-of-fit. Multinomial logistic regressions were run using the need for ventilatory support as a dependent variable and age, sex, obesity, cardiovascular disease, and diabetes as independent variables. Binary logistic regressions were run using the outcome (alive or death) or the need for intensive unit care (yes or no) as dependent variables and age, sex, obesity, cardiovascular disease, and diabetes as independent variables. For all regression models, the only continuous variable (age) was standardized (*Z*-score) to obtain the standardized odds ratios. Statistical analysis was conducted with SPSS v.20, and the adopted statistical significance level was 0.05.

Taking into consideration that in large samples, small effect sizes can be statistically significant [21], we also assess the following effect sizes to evaluate the magnitude of group differences and effects: eta squared (*η*^2^) for the Mann–Whitney and Wilcoxon tests, Cramer's V for the chi-square tests, and standardized odds ratio (OR) for the logistic regression models. Eta squared and Cramer's V were calculated according to Fritz and colleagues and Kim, respectively [22, 23]. Cohen's criteria were used to classify the effect sizes as small, medium, or large [22–24].

## 3. Results

### 3.1. Clinical and Demographic Characteristics of COVID-19 Mortality

Initially, we sought to describe the profile of the Brazilian population infected with COVID-19; a total of 182,700 patients infected with COVID-19 were included in this analysis. From the total confirmed deaths from COVID-19 (61,829), 58% were men and 74% of patients were 60 years of age or older. The highest numbers of deaths were among patients with cardiovascular disease (23,757), followed by diabetes (19,000), hypertension (8,475), chronic kidney disease (3,836), chronic neurological disease (3,476), pneumopathy (3,090), obesity (2,244), immunosuppression (2,161), cancer (2,155), and asthma (1,192) (Table 1).

### 3.2. Patients with Obesity Who Died of COVID-19 Are Younger than Patients without Obesity

We first considered the effect of age dichotomizing the cohort of patients in two age groups, < and ≥60 years old. As shown in Table 1, the proportion of deaths is significantly higher in the age group ≥60 years (74%). To further investigate the role of age and comorbidities in the death from COVID-19, we compared the frequency of deaths within each group of preexisting diseases, stratified by 60-year-old cutoffs, by using the goodness-of-fit chi-square. Since the sample number is large, the *P* value tends to be small (<0.05) even when the difference between the groups is minimal. Thus, Cramer's V effect size was also calculated. Considering the number of degrees of freedom in the analysis, Cramer's V is considered negligible below 0.1 [24]. Interestingly, among the variables analyzed, obesity was the only one for which no difference was present in the number of deaths between the two age groups. For all the other variables, the number of deaths was higher in the ≥60 years age group (Table 2).

Then, we compared the median age of patients who died of COVID-19 in each preexisting comorbidity (Table 3), also calculating the “eta squared” (*η*^2^), an effect size suitable for the Mann–Whitney test. In case *η*^2^ is between 0.01 and 0.06, the difference among the groups is considered small. If between 0.06 and 0.14, the difference is average, and above 0.14 is large [24]. Therefore, differences with *η*^2^ < 0.01 were considered nonsignificant.

A statistically significant difference in the age distribution was found for obesity, as the median age of patients with obesity was significantly lower (59 years (IQR = 23)) than that of subjects without obesity (71 years (IQR = 20), *P* < 0.001, and *η*^2^ = 0.0424). The same was found for immunosuppression (65 years (IQR = 22) vs. 71 years (IQR = 20), *P* < 0.001, and *η*^2^ = 0.0116). Conversely, the median age was higher in patients who had cardiovascular disease (73 years (IQR = 18) vs. 68 years (IQR = 22), *P* < 0.001, and *η*^2^ = 0.0230) and neurological disease (80 years (IQR = 16) vs. 70 years (IQR = 20), *P* < 0.001, and *η*^2^ = 0.0522) (Table 3).

**Figure 1:**
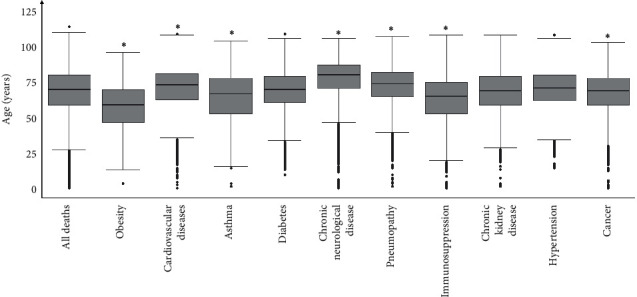
Comparison between the median age of death in the subgroups with comorbidity vs. the overall median age of death (70 years old). Obesity (*n* = 2244), cardiovascular disease (*n* = 23735), asthma (*n* = 1192), diabetes (*n* = 18986), neurological disease (*n* = 3470), pneumopathy (*n* = 3082), immunosuppression (*n* = 2154), chronic kidney disease (*n* = 3836), hypertension (*n* = 8475), cancer (*n* = 2155), and all deaths (*n* = 61829). One-sample Wilcoxon test.  ^*∗*^Eta squared (*η*^2^) > 0·01.

Our next step was to assess whether there is a difference between the median age of the total number of subjects who died of COVID-19 (70 years) and the median age of dead patients in each group of preexisting diseases. The Wilcoxon sign test was used, the “eta square” was applied as the effect size, and *η*^2^ < 0.01 corresponds to nonsignificant differences. We observed that patients with obesity (59 years (IQR = 23), *P* < 0.001, and *η*^2^ = 0.3518), asthma (67 years (IQR = 25), *P* < 0.001, and *η*^2^ = 0.0607), immunosuppression (65 years (IQR = 22), *P* < 0.001, and *η*^2^ = 0.1235), and cancer (69 years (IQR = 19), *P* < 0.001, and *η*^2^ = 0.0113) have age medians below the general median of 70 years (Figure 1 and Table S1). Conversely, the median age in cardiovascular disease (73 years (IQR = 18), *P* < 0.001, and *η*^2^ = 0.0227) and pneumopathy (74 years (IQR = 17), *P* < 0.001, and *η*^2^ = 0.0585) was higher than the median age of total cases (Figure 1 and Table S1). Among the comorbidities analyzed, obesity had the greatest effect size (*η*^2^ = 0.3518), which means that patients with obesity who died of COVID-19 had the highest difference in age compared to the median age of the total cohort of dead subjects (Table S1).

### 3.3. Women with Obesity Who Died of COVID-19 Are Older than Men with Obesity

**Figure 2:**
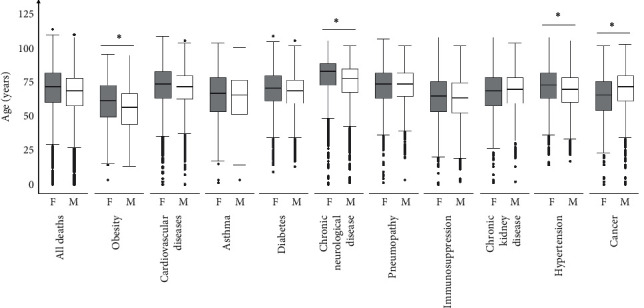
Comparison between men and women median age of death within each comorbidity subgroup and for all deaths. Obesity (*n* = 2244), cardiovascular disease (*n* = 23729), asthma (*n* = 1192), diabetes (*n* = 18979), neurological disease (*n* = 3470), pneumopathy (*n* = 3081), immunosuppression (*n* = 2153), chronic kidney disease (*n* = 3834), hypertension (*n* = 8472), cancer (*n* = 2155), and all deaths (*n* = 61671). F: females; M: males. Mann–Whitney test.  ^*∗*^Eta squared (*η*^2^) > 0·01.

Our next objective was to identify how COVID-19 deaths are distributed, considering the age and sex of the patients. First, we assessed whether the median age was different between men and women within each group of comorbidities by using Mann–Whitney's *U* test and calculating the “eta square.” Women have a median age higher than men when considering obesity, neurological disease, and hypertension ((55 years (IQR = 25) vs. 50 (IQR = 22), *P* < 0.001, and *η*^2^ = 0.0263), (80 years (IQR = 20) vs. 74 years (IQR = 22), *P* < 0.001, and *η*^2^ = 0.0333), and (67 years (IQR = 21) vs. 65 years (IQR = 20), *P* < 0.001, and *η*^2^ = 0.0114), respectively). However, for cancer patients, the median age of men was higher than that of women (70 years (IQR = 18) vs. 63 years (IQR = 23), *P* < 0.001, and *η*^2^ = 0.0301) (Figure 2 and Table S2).

In addition, we used the chi-square associated with Cramer's *V* to perform a statistical analysis of the frequency of deaths for each comorbidity. We found that the number of women with obesity who died in the age group ≥60 years was higher than expected and the number of men who died in the same age group was lower than expected (*P* < 0.001, Cramer's V = 0.1370) (Table 4). Conversely, in cancer patients, the number of women who died in the age group ≥60 years was lower than expected (*P* < 0.001, Cramer's V = 0.1480). No other disease showed a significant association between sex and age group (Table 4).

### 3.4. Obesity Is Associated with an Increase in ICU Need, Ventilatory Support, and Death of Hospitalized Patients with COVID-19

Through logistic regression analysis, we evaluated the influence of obesity as a risk factor for ventilatory support, intensive unit care (ICU) admission, and death. Obesity significantly increased the risk of using noninvasive (OR: 1.537; 95% CI: 1.414–1.670; *P* < 0.001) and even more invasive mechanical ventilatory support (OR: 2.302; 95% CI: 2.104–2.518; *P* < 0.001) (Table 5). Also, age increased the risk for both levels of ventilation needs (OR: 1.331; 95% CI: 1.301–1.363; *P* < 0.001 and OR: 1.504; 95% CI: 1.463–1.547; *P* < 0.001, for noninvasive and invasive, respectively) (Table 5). Besides, individuals with obesity are more likely to require ICU (OR: 1.783; 95% CI: 1.672–1.903; *P* < 0.001). Similarly, the risk of ICU admission increased with age (OR: 1.195; 95% CI: 1.171–1.220; *P* < 0.001) (Table 6). Both obesity and age also increased the patients' risk of dying of COVID-19 (OR: 1.411; 95% CI: 1.309–1.521; *P* < 0.001) and (OR: 2.354; 95% CI: 2.295–2.414; *P* < 0.001), respectively (Table 7). The risk of mechanical ventilatory support need (OR: 0.949; 95% CI: 0.911–0.989; and *P* 0.013 and OR: 0.806; 95% CI: 0.768–0.845; and *P* < 0.001, for noninvasive and invasive, respectively), ICU admission (OR: 0.842; 95% CI: 0.812–0.872; and *P* < 0.001), and death of COVID-19 (OR: 0.784; 95% CI: 0.753–0.816; and *P* < 0.001) is higher in men than in women (Tables 5–7). Furthermore, we evaluated the effect of the two most frequent preexisting comorbidities present in our cohort, cardiovascular disease and diabetes. Our results showed that the influence of cardiovascular disease and diabetes were less effective for COVID-19 severity than obesity (cardiovascular disease: OR: 1.203; 95% CI: 1.153–1.255; *P* < 0.001 for noninvasive and OR: 1.271; 95% CI: 1.210–1.335; *P* < 0.001 for invasive ventilatory support; OR: 1.161; 95% CI: 1.119–1.204; *P* < 0.001 for ICU, and OR: 0.975; 95% CI: 0.936–1.015; *P* 0.219 for death; diabetes: OR: 1.217; 95% CI: 1.166–1.270; *P* < 0.001 for noninvasive and OR: 1.389; 95% CI: 1.323–1.459; *P* < 0.001 for invasive ventilatory support; OR: 1.128; 95% CI: 1.088–1.169; *P* < 0.001 for ICU, and OR: 1.224; 95% CI: 1.176–1.274; *P* < 0.001 for death) (Tables 5–7).

## 4. Discussion

The development of more severe forms of COVID-19 in patients with obesity has been attributed to different mechanisms. The state of low-grade chronic inflammation characterizing obesity and the consequent development of immune dysfunction and insulin resistance, the high levels of angiotensin-converting enzyme (ACE2) produced by the expanded mass of adipose tissue, used by the virus to infect the host cells, the impaired lung function, and intestinal dysbiosis are among them. In addition, other obesity-associated comorbidities may also represent risk factors for COVID-19, such as diabetes, cardiovascular disease, and hypertension [19, 25–28].

Here, we showed that among the main diseases considered as a risk factor for COVID-19, obesity was the only one that showed no statistically significant difference in the number of deaths between patients stratified in <60 and ≥ 60 years of age (Table 2). Moreover, when considering the median age of the deaths, individuals with obesity died younger than individuals without obesity (Table 3) and had a median age lower than that of the total deaths, being the comorbidity where this difference was the most significant (Figure 1 and Table S1). Overall, our results indicate that obesity is an important risk factor for COVID-19 that seem to be more evident in the population under 60 years of age. These findings are consistent with other studies [12, 29, 30]. For instance, Kass and colleagues found an inverse correlation between age and BMI of patients in an American cohort of 265 patients with COVID-19 admitted to ICU; in this cohort, obesity was more diffuse in the younger individuals [30].

Similarly, analyzing data of COVID-19-positive patients from a hospital system in New York City, a study showed that among patients younger than 60 years, subjects with a BMI among 30–34 Kg/m^2^ were 2.0 times more likely to be admitted to acute care and 1.8 times more likely to be in critical care when compared to subjects with a BMI <30 Kg/m^2^. The risk of being admitted to acute care and critical care was raised to 2.2 and 3.6 times, respectively, for BMI >35 Kg/m^2^ in the same age group [12]. In another study performed in Wuhan (China) on patients with COVID-19 between 18 and 40 years of age, severe and critical cases had a significantly higher BMI than patients of moderate cases [29].

Thus, according to our data and supported by these findings in the literature, while age is an important risk factor for dying of COVID-19 in association with other comorbidities, this might not be true for obesity. Patients with obesity died of COVID-19 were younger than subjects without obesity, compared to the median age of all the other comorbidities considered in this study.

Considering sex, in our analysis, women with obesity had a significantly higher median age of death when compared to men (Figure 2 and Table S2). Also, comparing the frequency of COVID-19 dead men and women in the groups <60 and ≥ 60 years of age, we demonstrated that obesity and cancer were the only diseases that showed a significant difference between the observed and expected frequencies. Reinforcing our median analyses, the observed number of deaths of women with obesity in the age group ≥60 years was higher than expected (Table 4).

Previous studies have shown that the transmembrane protein ACE2 plays a key role in the pathogenesis of COVID-19, favoring the virus' entry into the host cell [31, 32]. Recently, its expression in the adipose tissue has been associated with the worst prognosis observed in patients with obesity affected by COVID-19 [33–35]. No difference in ACE2 expression has been observed between adipose tissue cells of individuals with and without obesity [36]. However, subjects with obesity have more adipose tissue than lean subjects and consequently more adipocytes with ACE2 receptors, resulting in a significant increase in the viral load and an exacerbation of the inflammatory status [36, 37]. ACE2 has been shown to be more expressed in the visceral adipose tissue (VAT) than in the subcutaneous fat [38], supporting the concept that the extent of the visceral adipose mass might be related to COVID-19 severity. Recently, Deng and colleagues found that this type of fat worsens the disease in young adults [29]. Another study showed that the amount of VAT is associated with a greater need for ICU and mechanical ventilation in patients with COVID-19 [39]. It is also important to note that excess adipose tissue, especially VAT, is associated with chronic low-grade inflammation present in obese individuals [40]. It is well established that metabolic disorders and the imbalance of the immune system resulting from this process play an important role in the relationship between obesity and other comorbidities [18]. Recently, some studies indicated these dysfunctions as responsible for developing more severe forms of COVID-19 in patients with obesity. However, the pathophysiological mechanisms underlying this putative relation have not yet been elucidated [27, 28].

During menopause, there is a reduction in estrogen and progesterone levels in women, which causes an increase in the extension of the visceral fat mass [41]. This increase in visceral fat mass might explain why women with obesity die of COVID-19 older than men. Also, a protective capacity of estrogen against disease development has been suggested as an advantage towards the male sex [42, 43].

Finally, according to our analysis, patients with obesity are more likely to be admitted to the ICU, to require mechanical ventilation, and to die of COVID-19 than individuals without obesity. In addition, our results showed that obesity is related to the severity of COVID-19 more than cardiovascular diseases and diabetes (Tables 5–7). These findings are in agreement with other studies present in the recent literature. The retrospective cohort study of patients admitted to intensive care for SARS-CoV-2 in a French hospital showed that individuals who needed invasive mechanical ventilation had a higher BMI than those who did not [10]. Also, the number of patients requiring invasive mechanical ventilation during hospitalization in intensive care was positively correlated with the BMI, reaching more than 85.7% in the BMI group ≥35 Kg/m^2^ [10]. Similarly, other studies performed on COVID-19 patients in different countries listed obesity as one of the main risk factors associated with hospitalization and the critical evolution of the disease [11, 44, 45].

We recognize that our study has some limitations. First, the high frequency of missing or ignored values. As it is a public database, adequate control over data filling cannot be guaranteed and much information about the presence or absence of comorbidities is absent or has been ignored. Second, unfortunately, there is no information in the database that would be relevant to discuss this study and understanding the development of severe forms of COVID-19, such as cytokine levels, inflammatory markers, and other laboratory indexes. Although a column for BMI values is present, we decided not to use it because we found the data inconsistent. Finally, it is essential to consider underreporting cases of COVID-19 in the country. Despite these points, our study has a significant number of patients and valuable information for a better understanding of some epidemiological aspects of the disease.

## 5. Conclusions

Despite the lower frequency of COVID-19 cases and deaths in obesity than other comorbidities, this pathology represents a pivotal factor associated with the critical evolution of the disease. We showed that COVID-19 patients with obesity die younger than individuals without obesity and women with obesity die older than men. These differences may be due to a different distribution of visceral adipose tissue and levels of sex steroid hormones, in particular, estrogens, characterizing obesity and sex. Also, in COVID-19 patients, obesity has been associated with an increased risk of ICU admittance, needs of mechanical ventilation, and death. It is essential to consider obesity as a warrant public health problem due to the significant impact of this disease in worsening pathologies as COVID-19. Additional effective actions and health/social policy more focused on fighting the obesity epidemic are urgently needed in the light of the tremendous impact of this chronic disease on COVID-19 and possible upcoming viral epidemics.

## Figures and Tables

**Table 1:** Overview of the COVID-19-infected patients divided by sex, age group, and preexisting disease.

	Alive			Death			Unknown		
	*n*	%	% valid cases	*n*	%	% valid cases	*n*	%	% valid cases
Sex									
Female	34729	44.67	44.68	25745	41.64	41.65	18837	43.68	43.7
Male	43001	55.31	55.32	36064	58.33	58.35	24272	56.28	56.3
Missing values	16	0.02	—	20	0.03	—	16	0.04	—

Age									
<60	50057	64.39	64.89	16135	26.1	26.15	24270	56.28	56.67
≥60	27080	34.83	35.11	45556	73.68	73.85	18559	43.04	43.33
Missing values	609	0.78	—	138	0.22	—	296	0.69	—

Obesity									
No	21293	27.39	88.99	20623	33.35	90.19	12266	28.44	88.51
Yes	2634	3.39	11.01	2244	3.63	9.81	1592	3.69	11.49
Missing values	53819	69.22	—	38962	63.02	—	29267	67.87	—

Cardiovascular disease									
No	12343	15.88	37.42	10230	16.55	30.1	6732	15.61	35.32
Yes	20642	26.55	62.58	23757	38.42	69.9	12327	28.58	64.68
Missing values	44761	57.57	—	27842	45.03	—	24066	55.81	—

Diabetes									
No	14949	19.23	49.38	12754	20.63	40.17	8156	18.91	46.16
Yes	15326	19.71	50.62	19000	30.73	59.83	9514	22.06	53.84
Missing values	47471	61.06	—	30075	48.64	—	25455	59.03	—

Asthma									
No	22173	28.52	90.58	22050	35.66	94.87	12901	29.92	92.07
Yes	2305	2.96	9.42	1192	1.93	5.13	1111	2.58	7.93
Missing values	53268	68.52	—	38587	62.41	—	29113	67.51	—

Pneumopathy									
No	22524	28.97	92.28	20925	33.84	87.13	12821	29.73	91.4
Yes	1884	2.42	7.72	3090	5	12.87	1206	2.8	8.6
Missing values	53338	68.61	—	37814	61.16	—	29098	67.47	—

Chronic neurological disease									
No	22670	29.16	92.71	20795	33.63	85.68	12867	29.84	91.27
Yes	1782	2.29	7.29	3476	5.62	14.32	1231	2.85	8.73
Missing values	53294	68.55	—	37558	60.74	—	29027	67.31	—

Chronic kidney disease									
No	22301	28.68	92.03	20397	32.99	84.17	12570	29.15	89.46
Yes	1930	2.48	7.97	3836	6.2	15.83	1481	3.43	10.54
Missing values	53515	68.83	—	37596	60.81	—	29074	67.42	—

Immunosuppression									
No	22406	28.82	92.56	21349	34.53	90.81	12820	29.73	92.08
Yes	1800	2.32	7.44	2161	3.5	9.19	1102	2.56	7.92
Missing values	53540	68.87	—	38319	61.98	—	29203	67.72	—

Hypertension									
Yes	7694	9.9	100	8475	13.71	100	4227	9.8	100
Missing values	70052	90.1	—	53354	86.29	—	38898	90.2	—

Cancer									
Yes	1155	1.49	100	2155	3.49	100	723	1.68	100
Missing values	76591	98.51	—	59674	96.51	—	42402	98.32	—

**Table 2:** Deaths by COVID-19 within preexisting diseases and age group.

	Age (years)			Χ^2^	*P*	Cramer's V
	<60	≥60	Total			
All deaths^^*∗*^^	16130	45541	61671	14031.14	<0.001	0.4770
Obesity	1124	1120	2244	0.007	0.933	0.0018
Cardiovascular disease^^*∗*^^	4167	19562	23729	9993.29	<0.001	0.6490
Diabetes^^*∗*^^	4175	14804	18979	5958.31	<0.001	0.5603
Asthma^^*∗*^^	414	778	1192	111.154	<0.001	0.3054
Pneumopathy^^*∗*^^	459	2622	3081	1519.43	<0.001	0.7023
Neurological disease^*∗*^	2355	8350	10705	2094.64	<0.001	0.4423
Immunosuppression^*∗*^	798	1355	2153	144.552	<0.001	0.2591
Chronic kidney disease^*∗*^	971	2863	3834	935.15	<0.001	0.4939
Hypertension^*∗*^	1694	6778	8472	3048.6	<0.001	0.5999
Cancer^*∗*^	563	1592	2155	491.342	<0.001	0.4775

Goodness-of-fit chi-square test. ^*∗*^Cramer's V > 0.1.

**Table 3:** Median age of death in subgroups with and without comorbidities.

	Median	IQR	Z	*P*	*N*	Eta squared
Obesity ^*∗*^	—	—	31.095	<0.001	22821	0.0424
Yes	59	23	—	—	—	—
No	71	20	—	—	—	—

Cardiovascular disease ^*∗*^	—	—	−27.926	<0.001	33929	0.0230
Yes	73	18	—	—	—	—
No	68	22	—	—	—	—

Asthma	—	—	7.876	<0.001	23196	0.0027
Yes	67	25	—	—	—	—
No	71	20	—	—	—	—

Diabetes	—	—	5.157	<0.001	31697	0.0008
Yes	70	18	—	—	—	—
No	71	22	—	—	—	—

Neurological disease ^*∗*^	—	—	−35.559	<0.001	24224	0.0522
Yes	80	16	—	—	—	—
No	70	20	—	—	—	—

Pneumopathy	—	—	−13.783	<0.001	23966	0.0079
Yes	74	17	—	—	—	—
No	70	21	—	—	—	—

Immunosuppression ^*∗*^	—	—	16.474	<0.001	23463	0.0116
Yes	65	22	—	—	—	—
No	71	20	—	—	—	—

Chronic kidney disease	—	—	3.46	<0.001	24817	0.0005
Yes	69	20	—	—	—	—
No	71	20	—	—	—	—

Sex	—	—	−23.486	<0.001	61671	0.0089
F	72	21	—	—	—	—
M	69	20	—	—	—	—

F: females; IQR: interquartile range; M: males. Mann–Whitney test.  ^*∗*^Eta squared (*η*^2^) > 0.01.

**Table 4:** Frequencies of death in men and women with comorbidities by age group.

	Age (years)			Χ^2^	*P*	Cramer's V
	<60	≥60	Total			
All deaths	—	—	—	193.205	<0.001	0.0560
F	5968	19711	25679			
M	10162	25830	35992			
Total	16130	45541	61671			

Obesity^*∗*^	—	—	—	41.728	<0.001	0.1370
F	470	622	1092			
M	654	498	1152			
Total	1124	1120	2244			

Cardiovascular disease	—	—	—	37.517	<0.001	0.0400
F	1647	8749	10396			
M	2520	10813	13333			
Total	4167	19562	23729			

Diabetes	—	—	—	47.129	<0.001	0.0500
F	1695	6899	8594			
M	2480	7905	10385			
Total	4175	14804	18979			

Asthma	—	—	—	0.023	0.879	0.0060
F	232	441	673			
M	182	337	519			
Total	414	778	1192			

Pneumopathy	—	—	—	5.742	0.017	0.0440
F	208	1029	1237			
M	251	1593	1844			
Total	459	2622	3081			

Neurological disease	—	—	—	14.885	<0.001	0.0660
F	154	1552	1706			
M	233	1531	1764			
Total	387	3083	3470			

Immunosuppression	—	—	—	0.499	0.48	0.0160
F	353	622	975			
M	445	733	1178			
Total	798	1355	2153			

Chronic kidney disease	—	—	—	6.972	0.008	0.0430
F	412	1076	1488			
M	559	1787	2346			
Total	971	2863	3834			

Hypertension	—	—	—	31.667	<0.001	0.0610
F	635	3057	3692			
M	1059	3721	4780			
Total	1694	6778	8472			

Cancer ^*∗*^	—	—	—	46.442	<0.001	0.1480
F	330	666	996			
M	233	926	1159			
Total	563	1592	2155			

F: females; M: males. Chi-square of independence test.  ^*∗*^Cramer's V > 0.1.

**Table 5:** Multinomial logistic regression with ventilatory support as the dependent variable and age, sex, obesity, cardiovascular disease, and diabetes as independent variables.

	Ventilatory support			
	Noninvasive vs. no		Invasive vs. no	
	OR (95% CI)	*P*	OR (95% CI)	*P*
Age (*Z*-score)^*∗*^	1.331 (1.301–1.363)	<0.001	1.504 (1.463–1.547)	<0.001
Gender (F vs. M)^*∗*^	0.949 (0.911–0.989)	0.013	0.806 (0.768–0.845)	<0.001
Obesity (yes vs. no)^*∗*^	1.537 (1.414–1.670)	<0.001	2.302 (2.104–2.518)	<0.001
Cardiovascular disease (yes vs. no)^*∗*^	1.203 (1.153–1.255)	<0.001	1.271 (1.210–1.335)	<0.001
Diabetes (Yes vs. no)^*∗*^	1.217 (1.166–1.270)	<0.001	1.389 (1.323–1.459)	<0.001

CI: confidence interval; F: females; M: males; OR: odds ratio. ^*∗*^, *P* < 0.05.

**Table 6:** Binary logistic regression with the need of ICU (yes vs. no) as the dependent variables and age, sex, obesity, cardiovascular disease, and diabetes as independent variables.

	ICU (yes vs. no)	
	OR (95% CI)	*P*
Age (*Z*-score)^*∗*^	1.195 (1.171–1.220)	<0.001
Gender (F vs. M)^*∗*^	0.842 (0.812–0.872)	<0.001
Obesity (yes vs. no)^*∗*^	1.783 (1.672–1.903)	<0.001
Cardiovascular disease (yes vs. no)^*∗*^	1.161 (1.119–1.204)	<0.001
Diabetes (yes vs. no)^*∗*^	1.128 (1.088–1.169)	<0.001

CI: confidence interval; F: females; M: males; ICU: intensive care unit; OR: odds ratio. ^*∗*^, *P* < 0.05.

**Table 7:** Binary logistic regression with the outcome (death vs. alive) as the dependent variables, and age, sex, obesity, cardiovascular disease, and diabetes as independent variables.

	Outcome (death vs. alive)	
	OR (95% CI)	*P*
Age (*Z*-score)^*∗*^	2.354 (2.295–2.414)	<0.001
Gender (F vs. M)^*∗*^	0.784 (0.753–0.816)	<0.001
Obesity (yes vs. no)^*∗*^	1.411 (1.309–1.521)	<0.001
Cardiovascular disease (yes vs. no)	0.975 (0.936–1.015)	0.219
Diabetes (yes vs. no)^*∗*^	1.224 (1.176–1.274)	<0.001

CI: confidence interval; F: females; M: males; and OR: odds ratio. ^*∗*^, *P* < 0.05.

## Data Availability

All data used to support the findings of this study are available from the corresponding author upon request.

## Supplementary Materials

Supplementary Materials Table S1. Comparison between the median age of death in the different comorbidity subgroups vs. the overall median age of death (70 years old). Table S2. Comparison between men and women median age of death within each comorbidity subgroup.
