# Supplementary material for: COVID-19 and Obesity: An Epidemiologic Analysis of the Brazilian Data
Source: Int J Endocrinol. 2021 May 5;2021:6667135. doi: 10.1155/2021/6667135 (PMC8121602; doi:10.1155/2021/6667135)
Supplement: Supplementary Materials — Table S1. Comparison between the median age of death in the different comorbidity subgroups vs. the overall median age of death (70 years old). Table S2. Comparison between men and women median age of death within each comorbidity subgroup. [file 6667135.f1.docx]

**COVID-19 and obesity: an epidemiologic analysis of the Brazilian data**

Diego Assis Gonçalves^1,2^, Victória Ribeiro^1^, Ana Gualberto^1^, Fernanda Fiel Peres^3^, Michaela Luconi^2,^* and Jacy Gameiro^1,^*

**Contact info:**

Diego de Assis Gonçalves

Department of Parasitology, Microbiology and Immunology

Federal University of Juiz de Fora

Juiz de Fora – MG, 36036-900, Brazil

diegoassisg@gmail.com

**Supplementary Table 1.** Comparison between the median age of death in the different comorbidity subgroups *vs.* the overall median age of death (70 years old).

|  | **Median** | **IQR** | **z** | ***P*** | **n** | **eta squared** |
| --- | --- | --- | --- | --- | --- | --- |
| **Obesity*** | 59 | 23 | -28.097 | < 0.001 | 2244 | 0.3518 |
| **Cardiovascular disease*** | 73 | 18 | 23.24 | < 0.001 | 23735 | 0.0227 |
| **Asthma*** | 67 | 25 | -8.507 | < 0.001 | 1192 | 0.0607 |
| **Diabetes** | 70 | 18 | -4.886 | < 0.001 | 18986 | 0.0012 |
| **Neurological disease*** | 80 | 16 | 28.541 | < 0.001 | 3470 | 0.2347 |
| **Pneumopathy*** | 74 | 17 | 13.436 | < 0.001 | 3082 | 0.0585 |
| **Immunosuppression*** | 65 | 22 | -16.311 | < 0.001 | 2154 | 0.1235 |
| **Chronic kidney disease** | 69 | 20 | -4.494 | < 0.001 | 3836 | 0.0052 |
| **Hypertension** | 71 | 18 | 5.756 | < 0.001 | 8475 | 0.0039 |
| **Cancer*** | 69 | 19 | -4.935 | < 0.001 | 2155 | 0.0113 |

IQR, interquartile range. One-sample Wilcoxon test. * for eta squared (η^2^) > 0.01.

**Supplementary Table 2.** Comparison between men and women median age of death within each comorbidity subgroup.

|  | **Median** | **IQR** | **z** | ***P*** | **n** | **eta squared** |
| --- | --- | --- | --- | --- | --- | --- |
| **Obesity*** | | | -7.679 | < 0.001 | 2244 | 0.0263 |
| F | 55 | 25 |  |  |  |  |
| M | 50 | 22 |  |  |  |  |
| **Cardiovascular Disease** | | | -14.787 | < 0.001 | 23729 | 0.0092 |
| F | 69 | 22 |  |  |  |  |
| M | 67 | 20 |  |  |  |  |
| **Asthma** | | | -0.969 | 0.332 | 1192 | 0.0008 |
| F | 55 | 29 |  |  |  |  |
| M | 52 | 28 |  |  |  |  |
| **Diabetes** | | | -12.407 | < 0.001 | 18979 | 0.0081 |
| F | 67 | 20 |  |  |  |  |
| M | 65 | 19 |  |  |  |  |
| **Neurological disease *** | | | -10.744 | < 0.001 | 3470 | 0.0333 |
| F | 80 | 20 |  |  |  |  |
| M | 74 | 22 |  |  |  |  |
| **Pneumopathy** | | | 0.657 | 0.511 | 3081 | 0.0001 |
| F | 70 | 22 |  |  |  |  |
| M | 71 | 19 |  |  |  |  |
| **Immunosuppression** | | | -1.01 | 0.312 | 2153 | 0.0005 |
| F | 59 | 27 |  |  |  |  |
| M | 59 | 25 |  |  |  |  |
| **Chronic kidney disease** | | | 1.843 | 0.065 | 3834 | 0.0009 |
| F | 66 | 24 |  |  |  |  |
| M | 67 | 20 |  |  |  |  |
| **Hypertension*** | | | -9.826 | < 0.001 | 8472 | 0.0114 |
| F | 67 | 21 |  |  |  |  |
| M | 65 | 20 |  |  |  |  |
| **Cancer*** | | | 8.048 | < 0.001 | 2155 | 0.0301 |
| F | 63 | 23 |  |  |  |  |
| M | 70 | 18 |  |  |  |  |
| **All deaths** | | | -23.486 | < 0.001 | 61671 | 0.0089 |
| F | 72 | 21 |  |  |  |  |
| M | 69 | 20 |  |  |  |  |

F, females; M, males. Mann-Whitney test. * for eta squared (η^2^) > 0.01.
